# The effects of different daily irradiance profiles on Arabidopsis growth, with special attention to the role of PsbS

**DOI:** 10.3389/fpls.2023.1070218

**Published:** 2023-03-09

**Authors:** Christo Schiphorst, Cas Koeman, Ludovico Caracciolo, Koen Staring, Tom P. J. M. Theeuwen, Steven M. Driever, Jeremy Harbinson, Emilie Wientjes

**Affiliations:** ^1^ Laboratory of Biophysics, Wageningen University & Research, Wageningen, Netherlands; ^2^ Laboratory of Genetics, Wageningen University & Research, Wageningen, Netherlands; ^3^ Centre for Crop Systems Analysis, Wageningen University & Research, Wageningen, Netherlands

**Keywords:** fluctuating light, photosynthesis, CO_2_ assimilation, leaf area, Arabidopsis

## Abstract

In nature, light is never constant, while in the controlled environments used for vertical farming, *in vitro* propagation, or plant production for scientific research, light intensity is often kept constant during the photoperiod. To investigate the effects on plant growth of varying irradiance during the photoperiod, we grew *Arabidopsis thaliana* under three irradiance profiles: a square-wave profile, a parabolic profile with gradually increasing and subsequently decreasing irradiance, and a regime comprised of rapid fluctuations in irradiance. The daily integral of irradiance was the same for all three treatments. Leaf area, plant growth rate, and biomass at time of harvest were compared. Plants grown under the parabolic profile had the highest growth rate and biomass. This could be explained by a higher average light-use efficiency for carbon dioxide fixation. Furthermore, we compared the growth of wild type plants with that of the PsbS-deficient mutant *npq4*. PsbS triggers the fast non-photochemical quenching process (qE) that protects PSII from photodamage during sudden increases in irradiance. Based mainly on field and greenhouse experiments, the current consensus is that *npq4* mutants grow more slowly in fluctuating light. However, our data show that this is not the case for several forms of fluctuating light conditions under otherwise identical controlled-climate room conditions.

## 1 Introduction

In nature, the irradiance incident on a leaf changes over the course of a day. These fluctuations occur on multiple timescales, ranging from a second to minutes for sunflecks caused by air movement moving leaves higher in the canopy or by cloud movement, to fluctuations caused by cloud movement lasting between minutes and hours, to the diurnal change in irradiance as the sun rises and sets as a result of the rotation of the Earth around its axis (Pearcy, 1990; Ruban, 2009; Kaiser et al., 2018; Wang et al., 2020). In low light, when photosynthesis is light-limited, plants must absorb as much light as possible for photosynthesis and use it as efficiently as possible. In contrast, in high light, when photosynthesis is light-saturated, more energy is absorbed than can be used for photosynthesis. If left unchecked, this excess of energy can actually damage the plant. As a result of these changing priorities, plants must constantly maintain a balance between efficient photosynthesis in low light and photoprotection in high light (Pearcy, 1990; Ruban, 2009; Kaiser et al., 2018; Wang et al., 2020; Long et al., 2022). Matters are complicated in cases of fluctuating irradiance because an increase in irradiance (provided that assimilation is not already light-saturated) will produce an increase in assimilation, which results in a decrease in the degree of excess of irradiation. As a result, the degree to which irradiance is in excess changes (and normally decreases) with time.

Plants have developed multiple ways to respond to changes in light intensity (Ruban, 2009; Murchie and Niyogi, 2011; Kaiser et al., 2018). A major adaptation mechanism is the circadian rhythm, based on the oscillating day–night cycle of terrestrial daylight. It is estimated that 25-35% of the *Arabidopsis thaliana* (Arabidopsis) genome is controlled by the circadian rhythm (Covington and Harmer, 2007; Hazen et al., 2009). Unsurprisingly, photosynthesis is also influenced by circadian oscillations *via* various pathways and mechanisms (Dodd et al., 2014). This is reflected in the fact that photosynthesis, as assessed by net CO_2_ assimilation rate (*A_net_*) and stomatal conductance, continues to display a circadian rhythm in plants exposed to constant light (Hennessey and Field, 1991). As plants have evolved under a natural daytime light regime, in which potential irradiance increases gradually until noon and then decreases until sunset, it can be hypothesized that plants should be adapted to this irradiance profile and thus should grow more quickly under a natural, approximately parabolic (or sinusoidal) irradiance profile than under square-wave (on/off) light conditions. Knowledge of such an adaptation would be important in guiding the control of irradiance in vertical farming, where crops such as lettuce are grown indoors under light-emitting diode lamps (LEDs) (van Delden et al., 2021). An increase in plant biomass produced per unit kWh of electricity used for lighting would provide an economic advantage. A hint that sinusoidal light does provide an advantage comes from the work of Chiang et al. (2020), which shows that the leaf area of several species is larger for plants grown under sinusoidal light than for plants grown under square-wave light conditions with the same daily integral of irradiance.

Rapid fluctuations in light intensity, on the timescale of seconds to minutes, are very common in the understory of forests and in the canopy of densely packed crops growing in the field (Pearcy, 1990; Ruban, 2009; Kaiser et al., 2018; Wang et al., 2020; Long et al., 2022). Such fluctuations are challenging for plants and have been shown to negatively affect plant growth and fitness (Kulheim et al., 2002; Alter et al., 2012; Poorter et al., 2016; Vialet-Chabrand et al., 2017; Kaiser et al., 2018; Qiao et al., 2021). The main mechanism of protection against sudden high light is qE, or energy-dependent non-photochemical quenching (NPQ), the process in PSII that underlies the protective conversion to heat of those excited states of chlorophyll that are in excess of the needs of photochemistry. Excess irradiance above the requirements of photosynthetic metabolism leads to acidification of the thylakoid lumen, which is sensed by the protein PsbS and catalyzes the quenching of excited states, thereby giving rise to the phenomenon of qE (Li et al., 2000; Li et al., 2002). The enzymatic conversion of the carotenoid violaxanthin into zeaxanthin further amplifies qE (Demmig-Adams, 1990; Niyogi et al., 1998). The establishment and relaxation of qE is slow relative to the more rapid fluctuations of irradiance encountered in the field (seconds or tens of seconds for qE versus seconds or less for irradiance fluctuations). Based on *in silico* experiments, the slow relaxation of qE, which can limit the light-use efficiency of PSII electron transport for photosynthesis, has been proposed to be potentially a limiting factor for photosynthesis and crop carbon gain (Zhu et al., 2004). Accelerating the relaxation of qE *via* over-expression of PsbS and the enzymes involved in the reversible conversion of violaxanthin into zeaxanthin has been found to result in increased crop productivity in the field in tobacco plants (Kromdijk et al., 2016) and increased crop yield in soybean (De Souza et al., 2022). On the other hand, the same mutations have been found to impair growth rate in Arabidopsis (Garcia-Molina and Leister, 2020). It is generally believed that lacking PsbS negatively affects plant performance under light fluctuations: a PsbS knock-out, known as *npq4*, produces fewer seeds (Kulheim et al., 2002; Krah and Logan, 2010), has a reduced leaf area (Logan et al., 2008; Krah and Logan, 2010), and exhibits reduced CO_2_ assimilation (Hubbart et al., 2012). However, under constant irradiance during the photoperiod, a lack of PsbS does not seem to confer any disadvantages (Kulheim et al., 2002; Khuong et al., 2019) and could even represent an advantage under constant low irradiance (Khuong et al., 2019).

Thus far, most fluctuating light studies on the *npq4* mutant have been performed under uncontrolled field conditions or in greenhouses. As such, it is unclear under which kind of light fluctuations possession of PsbS is required for optimal plant growth and biomass production. If we are to engineer plants with improved photosynthetic efficiency for higher crop yields (Zhu et al., 2010), it is important to understand under which light conditions photoprotective quenching is beneficial for plant growth. A similar question could be asked for Stn7, the kinase of the major light-harvesting complex II that restores the balance of excitation of photosystems I and II under certain conditions of imbalance and thus improves the light-use efficiency of assimilation (Bellafiore et al., 2005; Taylor et al., 2019). It has been shown that absence of this protein also diminishes plant fitness and growth under fluctuating light conditions (Kulheim et al., 2002; Frenkel et al., 2007; Tikkanen et al., 2010; Grieco et al., 2012).

Several studies have investigated the effects of fluctuating light on plant growth, e.g., through use of a square-wave irradiance profile (Tikkanen et al., 2010), fluctuations that mimic a measured natural daytime light profile (Vialet-Chabrand et al., 2017; Chiang et al., 2020), or a natural increasing and decreasing intensity profile with added random fluctuations (Ferroni et al., 2020; von Bismarck et al., 2022). Here we have investigated the effects of different light regimes on the growth rate and biomass production of Arabidopsis plants. We compared the effects on wild type (WT) plants, *stn7* plants (lacking Stn7), and *npq4* plants. This comparison produced two interesting results. First, when they were grown under fluctuating light that mimics natural light conditions, the relative growth rate and above-ground biomass production of *npq4* and *stn7* plants were not significantly reduced compared to those of WT plants. Second, growing plants of each of these genotypes under a parabolic irradiance profile, resembling the natural diurnal increase and decrease in light intensity, resulted in enhanced biomass production. To investigate this further, we studied the effect of different fluctuating light conditions and temperatures on the growth of *npq4* plants compared to WT plants. Finally, the CO_2_ assimilation rate of WT plants was compared for the square-wave and parabolic irradiance regimes.

## 2 Materials and methods

### 2.1 Growth conditions

*A. thaliana* plants, accession *Columbia*, from lines WT, *npq4* (Li et al., 2000), and *stn7* (Bellafiore et al., 2005), were grown in controlled conditions of 24°C during the day and 20°C during the night, under a short-day light regime of 8 hours light and 16 hours darkness, with a light intensity of 125 µmol m^-2^ s^-1^. Seeds were allowed to germinate for 10 days before being transplanted into individual pots, where they were grown for another week before the experiments were started.

### 2.2 Growth under square-wave, parabolic, and fluctuating light conditions

Growth irradiance was provided by an LED array (Fluence Vypr 2p, Fluence Europe, Rotterdam, the Netherlands). The light intensity provided by this array is linearly dependent on the supply output current generated by the LED power supply. This output current was controlled using the dimmer function of the supply, which was linearly dependent on the value of a resistor placed between the dimmer control pins. Adjustable resistance between these pins was provided by an optocoupled light-dependent resistor actuated by a microcontroller (ESP32 – Espressif Systems; https://www.espressif.com). This digital controller allowed the irradiance to be altered every 3 seconds, this limit on the rate being set by the control electronics of the LED power supply.

**Figure 1:**
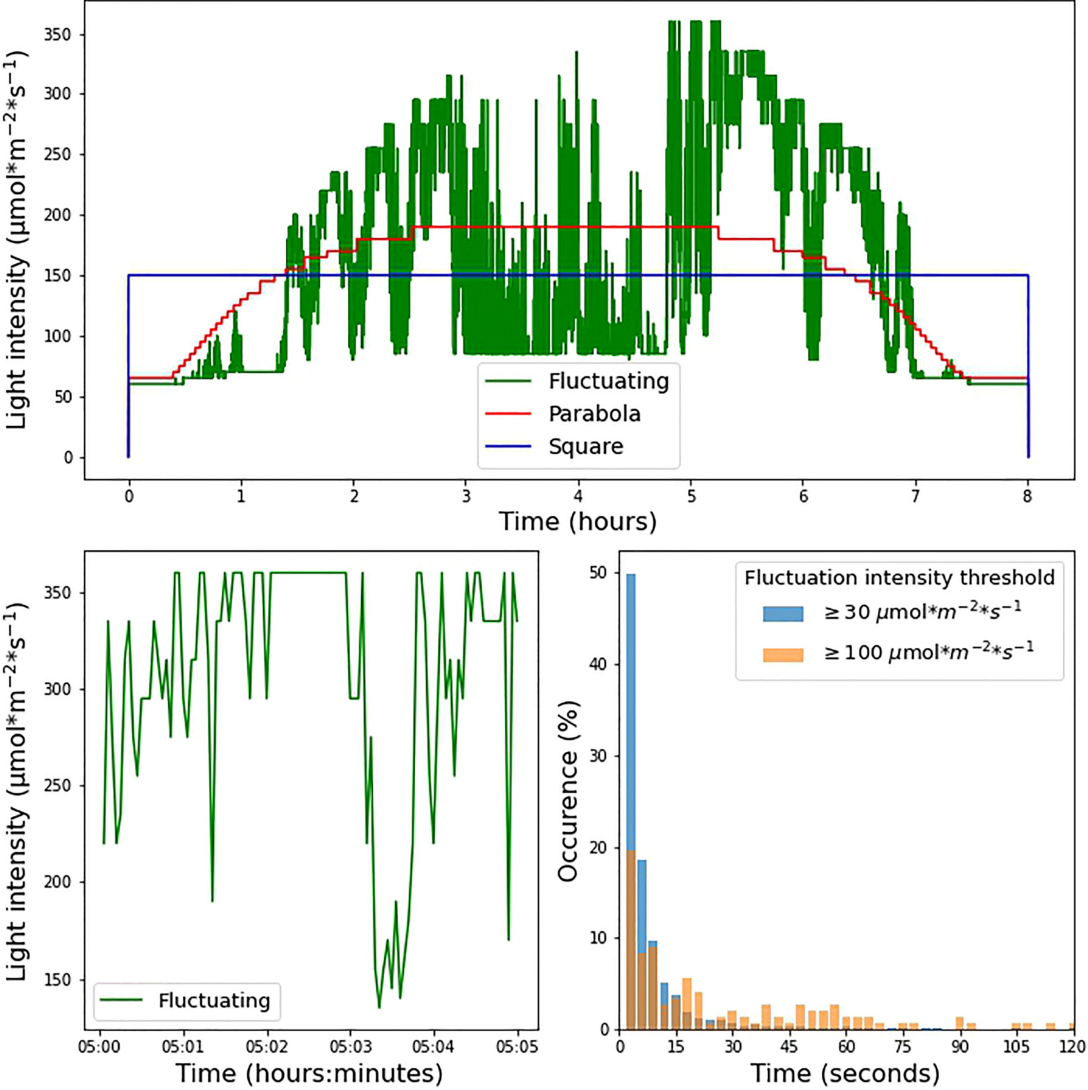
**(A)** Experimental light intensity conditions during the 8-hour photoperiod under which the plants were grown. **(B)** Zoomed-in visualization of 5 min under the fluctuating light condition. **(C)** Incidence of fluctuations in the fluctuating light condition. The histograms show the proportion of instances in which light intensity remained within a range of 30 µmol m^-2^ s^-1^ (blue bars) and 100 µmol m^-2^ s^-1^ (orange bars) for a given amount of time.

For the square wave, a continuous light intensity of 150 µmol m^-2^ s^-1^ was used throughout the photoperiod. The parabolic profile was interpolated from irradiance data based on measurements made available at solcast.com. The cloudless irradiance values used for this purpose were measured on 2021-11-11 at 21.9028° N, 12.4964° E, a location in the Sahara in Niger. The fluctuating light condition was based on measurements made on September 20^th^ 2020 in Wageningen (the Netherlands, 51°59’20.0”N, 5°39’43.2”E) 1.5m above ground in a mature maize canopy. Using a Licor quantum sensor, a laboratory-built transimpedance amplifier, and a Picolog ADC-24 datalogger, an irradiance dataset with 100 ms resolution was recorded. The average over 3-second intervals was used for the fluctuating condition, adjusted for the 8-hour photoperiod by taking a 2-hour slice of data from the middle of the day. Irradiance levels between 0 and 60 µmol m^-2^ s^-1^ could not be achieved by our system owing to limitations in the control of the LED power supply. As a result, both the parabolic profile and fluctuating light profile began with a stepwise increase. The three different illumination conditions were normalized to the same total daily integrated photosynthetic photon flux density (PPFD) over an 8-hour photoperiod (**Figure 1**).

In order to image the growth of plants in the growth cabinet, a Raspberry Pi device connected to 6 different USB webcams was programmed to collect images multiple times per day. The images were first corrected for fish-eye distortion using the Python module OpenCV. Subsequently, the coordinates of every individual plant pot were measured using ImageJ, which allowed the images to be sliced to form sub-images, each containing a single plant, and the area of each plant was measured. Leaf area was determined by converting the RGB image to the CIELAB color space, where the a* channel was inverted and converted to a mask before the leaf areas were automatically selected using an ImageJ script. Growth in leaf area (*A*) for each individual plant was then fitted using an expolinear growth model (Goudriaan and Monteith, 1990):


$$A=\frac{C_{m}}{R_{m}}ln\left(1+e^{R_{m}*\left(t-t_{b}\right)}\right)$$

where *R_m_* is the maximum relative growth rate in the exponential phase, *C_m_* is the maximum relative growth rate in the linear phase, *t* is elapsed time, and *t_b_* is the time at which the linear phase starts.

### 2.3 Measurement of CO_2_ assimilation using a custom-built system

CO_2_ assimilation measurements were performed as described in Taylor et al. (2019) using an LI-7000 CO_2_/H_2_O analyzer (LI-COR, NE, USA) operating in differential mode. The gas mix used for the measurements contained 400 µmol CO_2_ mol^-1^ (400ppm CO_2_), 200 mmol O_2_ mol^-1^ (20% oxygen), and 18.8 mmol H_2_O mol^-1^, and the remainder of the gas mix consisted of N_2_. The gas stream was divided between the reference cell of the gas analyzer and a custom-made leaf chamber, after which the gas stream was supplied to the analysis cell of the gas analyzer. The leaf chamber allowed an entire Arabidopsis leaf to be enclosed within the chamber *via* the petiole. The upper transparent window of the chamber was sealed against the metal rim of the lower half of the leaf chamber by a hard rubber O-ring coated with silicone grease, forming a gas-tight seal with no diffusive leaks.

An LED array was fitted on top of the leaf chamber; this was controlled by a constant-current LED driver (Mean Well LCM-40, Haarlem, the Netherlands) capable of rapid changes in current output. This driver was controlled by an ESP32 microcontroller *via* an optocoupler with a 2s interval in the case of the simulated parabolic irradiance profile.

Gas exchange measurements were performed on 5-week-old plants grown under square wave irradiance of 125 µmol m^-2^ s^-1^ during the photoperiod. Leaves were adapted for 15 minutes after being placed in the leaf chamber before the measurement was started.

CO_2_ assimilation was calculated by correcting for gas dilution by H_2_O released by the leaf using the following formula:


$$J_{CO_{2}}=\frac{J_{gas_{in}}}{A_{leaf}}\left(x_{CO_{2_{in}}}-x_{CO_{2_{out}}}\left(\frac{1-x_{H_{2}O_{in}}}{1-x_{H_{2}O_{out}}}\right)\right)$$

where *J*_*gas*_*in*__ is the total gas influx, *A_leaf_* is the total leaf area in the chamber, and *x* is the molar fraction of the respective gas measured at the influx or the outflux of the leaf chamber. Transpiration was calculated according to the following formula:


$$J_{H_{2}O}=\frac{J_{gas_{in}}}{A_{leaf}}*\left(\frac{x_{H_{2}O_{out}}-x_{H_{2}O_{in}}}{1-x_{H_{2}O_{out}}}\right)$$

### 2.4 Combined measurement of CO_2_ assimilation and chlorophyll fluorescence

Combined measurements of CO_2_ assimilation and chlorophyll fluorescence were taken for individual leaves using an open infrared gas-exchange system (LI-6400XT; LI-COR, Lincoln, NE) and a 2-cm^2^ leaf chamber with an integral blue–red LED light source and fluorometer (LI-6400–40; LI-COR, Lincoln, NE). Plants were dark acclimated and then exposed to three cycles of approximately 5 min of low light (100 µmol m^-2^ s^-1^) and 1 min of high light (1000 µmol m^-2^ s^-1^), followed by three cycles of 5 min of low light and 5 min of high light. Light supplied was a combination of red and 10% blue light. The operating efficiency of PSII electron transport (*Φ*_PSII_) was determined as (*F*_m_ *’*– *F′*)/*F*_m_ *’* (Genty et al., 1989), where *F*′ is the steady-state fluorescence and *F*_m_ *’* is the maximum fluorescence during the saturating light pulse, as determined by the multiphase flash method (Loriaux et al., 2013). The level of non-photochemical quenching (NPQ) was determined as (*F*_m_ – *F*_m_ *′*)/*F*_m_ *′*, where *F*_m_ is the maximum fluorescence in the dark-acclimated state and *F*_m_ *’* is the maximum fluorescence during the light-adapted state, both as determined by a multiphase flash (after (Loriaux et al., 2013); total duration was 0.9 seconds (0.3 seconds per phase), the ramp rate was 40%, and the maximum flash intensity was ~ 6000 µmol m^-2^ s^-1^). Conditions in the leaf cuvette were maintained at a CO_2_ concentration of 400 ppm, a VPD of approximately 1 kPa, and a leaf temperature of 25°C. Recordings of gas exchange and chlorophyll fluorescence were made every minute for the duration of the measurement period.

### 2.5 PSII quantum efficiency measurements

The ratio of maximum variable to maximum total Chl *a* fluorescence (*F*_v_/*F*_m_), determined after 30 min dark-adaptation, served as a measure of PSII quantum efficiency. Fluorescence measurements were performed with a PAM-101 fluorometer (Walz, Effeltrich, Germany). F_m_ was measured as the maximum fluorescence during a saturating pulse of 0.8 seconds with an intensity of ~6000 µmol m^-2^ s^-1^.

### 2.6 Fluctuating light; WT vs *npq4*

A lighting system was created in a plant growth cabinet using LED bars for the low-irradiance conditions (100 µmol m^-2^ s^-1^, LL) combined with an additional 4 high-power 3W LEDs with a 15° focusing lens to provide the high-irradiance (1000 µmol m^-2^ s^-1^, HL) conditions. Plants were grown under a short-day light regime of 8 hours light and 16 hours darkness at three different temperatures (4°C, 10°C, or 24°C). The temperature was unchanged throughout the day/night cycle. The switch to the high-power LEDs between LL and HL was managed using a relay controlled by a programmable Arduino microcontroller (https://www.arduino.cc). Three different conditions were programmed: 1h HL (1000 µmol m^-2^ s^-1^) and 30 min LL (100 µmol m^-2^ s^-1^); 1 min HL and 5 min LL; and 5 min HL and 5 min LL.

Growth was monitored by taking photographs of the plants (including a minimum of 10 plants for each genotype and each condition) every 3 or 4 days and counting the pixels for each plant using Adobe Photoshop CS6, using a 1-euro coin as a size reference in the images. After each experiment, the fresh above-ground weight of the plants was determined.

## 3 Results

### 3.1 The effect of square-wave, parabolic, and fluctuating light conditions on plant growth

We investigated the effects of different light regimes on the growth of WT, *npq4*, and *stn7* Arabidopsis plants. After 17 days of growth under continuous light (125 µmol m^-2^ s^-1^, 8-hour photoperiod), the plants were exposed to three different light conditions, all with an 8-hour photoperiod and the same daily integral of photosynthetic photon flux density (PPFD). The conditions were: 1) square-wave irradiance of 150 µmol m^-2^ s^-1^; 2) parabolic irradiance ranging from 65 to 190 µmol m^-2^ s^-1^, resembling the natural increase and decrease in light intensity during the day; and 3) rapidly fluctuating irradiance ranging from 60 to 360 µmol m^-2^ s^-1^, based on the measurements of light intensity fluctuations in a maize canopy in the field. The three light intensity profiles are shown in **Figure 1A** while **Figure 1B** shows a zoomed-in view of the fluctuating light profile (condition 3 above). We analyzed the changes imposed under the fluctuating light regime; **Figure 1C** shows the distribution of the time taken for the light intensity to change by ≥ 30 µmol m^-2^ s^-1^ or ≥ 100 µmol m^-2^ s^-1^. This analysis shows that periods of constant irradiance lasting 3 seconds occurred most frequently (this was the shortest time-interval over which the intensity was changed), while periods of constant irradiance lasting up to 1 minute were frequent. Periods of constant light intensity lasting more than 2 minutes were uncommon (1.8% of the total for changes ≥ 30 µmol m^-2^ s^-1^ and 10.5% of the total for changes ≥ 100 µmol m^-2^ s^-1^).

**Figure 2:**
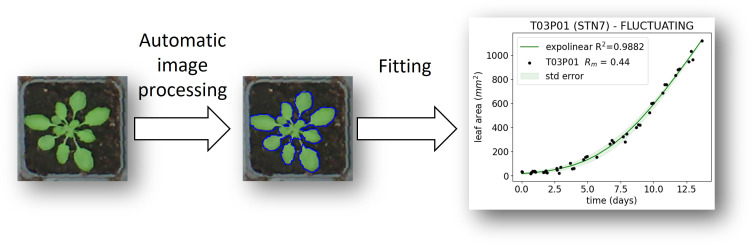
Workflow for analysis of increase in leaf area over time.

**Figure 3:**
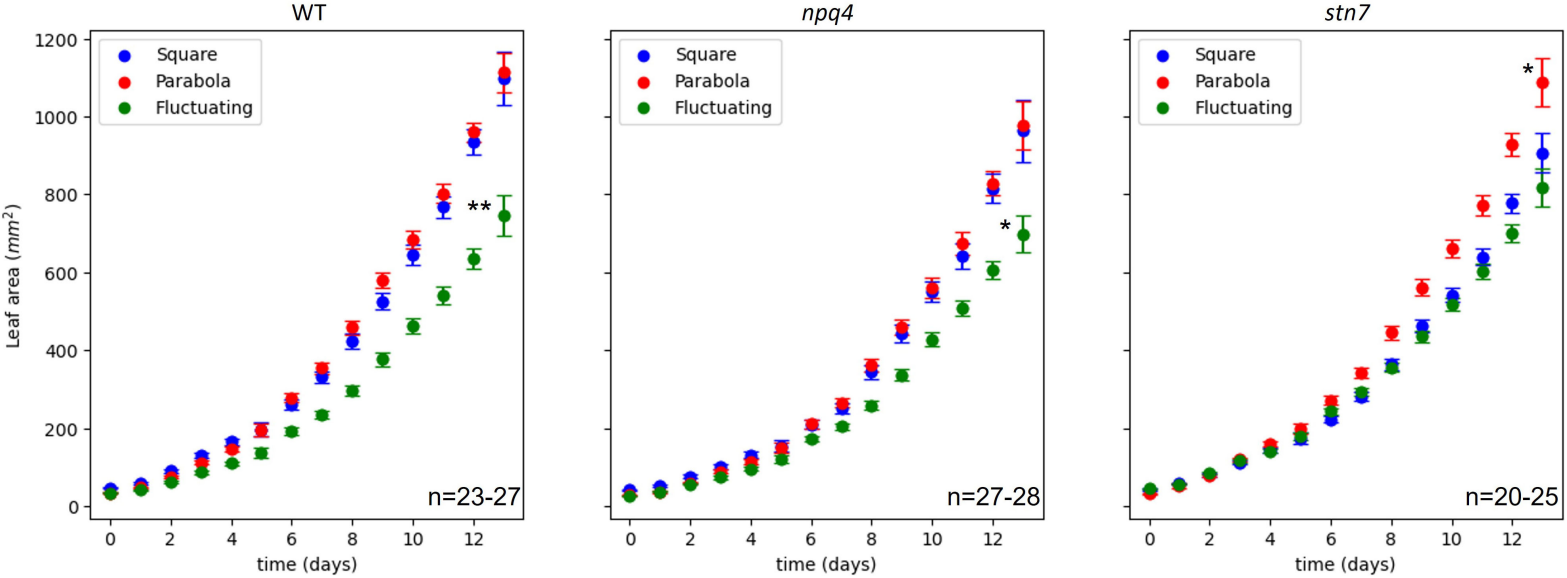
Increase in leaf area (mm^2^) during growth of WT, *npq4*, and *stn7* Arabidopsis plants under square-wave, parabolic, and fluctuating light conditions. The SE is shown. The number of plants (n) analyzed is displayed on each graph. Differences in final leaf area were tested via one-way ANOVA, followed by a Tukey HSD test. Significantly different values are indicated by asterisks, * for p<0.05 and ** for p<0.01.

In order to evaluate plant growth (**Supplementary Movie 1**), at least 3 images of the plants were collected each day, and the projected leaf area was measured using these images (**Supplementary Movie 2**). Based on these data, we plotted the increase in leaf area over time (**Figure 2**). Treating increase in leaf area as a metric for overall growth, we found that fluctuating irradiance significantly (*p*< 0.05) reduced plant growth in WT and *npq4* plants, compared to the two other conditions (**Figure 3**). For *stn7* plants, growth was fastest under a parabolic irradiance profile; the other two conditions (fluctuating and square) did not differ significantly from each other in terms of growth speed, but in both cases this was slower than under the parabolic profile.

**Figure 4:**
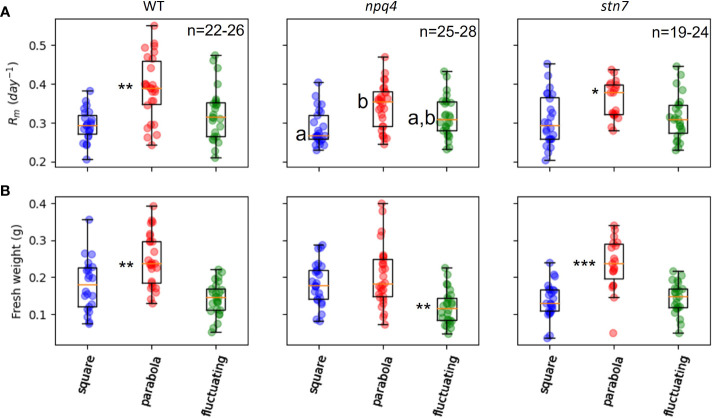
Maximal exponential growth rate **(A)** and fresh weight **(B)** of WT, npq4, and stn7 plants under square, parabolic, and fluctuating light conditions. Measurements are displayed in boxplot form. The middle line represents the median value of the data; values inside the box represent 50% of the measured data; and the whiskers together with the box comprise 95% of the measured values. Differences were tested for via one-way ANOVA, followed by a Tukey HSD test. Significantly different values are indicated by asterisks: * for p<0.05, ** for p<0.01, *** for p<0.001. For npq4 plants, the Rm values for the square and parabolic light conditions are significantly different (p<0.001), while there is no significant difference between the square and fluctuating conditions (p=0.122) or between the parabolic and fluctuating conditions (p=0.166).

Next, in order to analyze the effect of different irradiance profiles on the rate of plant growth in greater detail, growth in projected leaf area as function of time was parameterized by fitting an expolinear growth model (see Materials and Methods). Plant growth was initially exponential, but became linear as the canopy began to close (i.e., when the leaves began to overlap). We found that while *R_m_* (the maximum relative growth rate) could be estimated reliably from our data, due to the relatively short duration of this experiment, the uncertainty was large for the estimated values of *C_m_* (the maximum growth rate in the linear phase); therefore, this parameter was not used for further analysis. **Figure 4A** shows the *R_m_* values for the WT, *npq4*, and *stn7* plants under each of the three light conditions. Both WT and *stn7* plants showed a significantly higher R_m_ under a parabolic irradiance profile compared to the two other irradiance conditions. In contrast, *npq4* plants exhibited a significantly higher maximum relative growth rate under the parabolic irradiance than under the square-wave condition, but there were no significant differences in relative growth rate between fluctuating irradiance and the other two irradiance profiles. The total above-ground fresh weight of the plants at the end of the experiment showed a similar trend. Fresh weight was significantly higher for WT and *stn7* plants grown under a parabolic irradiance profile; *npq4* plants also had a higher fresh weight under parabolic irradiance compared to the other two profiles, but in this case the difference was not significant. For *npq4* plants, fresh weight was significantly lower when they were grown under fluctuating light conditions compared to square-wave and parabolic irradiance profiles. Finally, WT plants grown under fluctuating irradiance also had a fresh weight lower than those grown under parabolic or square-wave profiles, but in this case the difference was not significant.

Taken together, the data on leaf area and fresh weight from WT, *npq4*, and *stn7* plants showed similar overall trends in terms of the effects of different irradiance profiles on plant growth (**Figures 3**, **4**). Growth (as indexed by *R_m_* and fresh weight) was greatest under the parabolic profile condition relative to the square-wave and fluctuating profiles; note that a square-wave profile is conventionally used in controlled-environment systems.

### 3.2 Under which fluctuating light conditions does the absence of PsbS or Stn7 result in a growth disadvantage?

**Figure 5:**
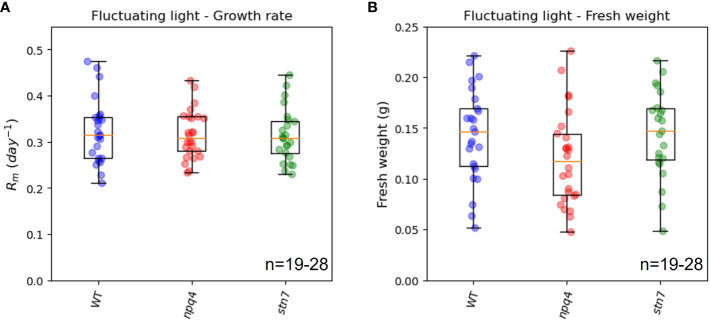
Exponential growth rate **(A)** and above-ground fresh weight **(B)** of WT, *npq4*, and *stn7* plants grown under fluctuating light. Measurements are displayed in boxplot form. The middle line represents the median value of the data; values inside the box represent 50% of the measured data; and the whiskers together with the box comprise 95% of the measured values. The number of plants (n) is indicated in the figure. The differences between the genotypes were not significant, p>0.1.

We compared the maximum relative growth rates (*R_m_*, **Figure 5A**), fresh weights (**Figure 5B**), and increases in leaf area (**Supplementary Figure 1**) of WT plants, *npq4* plants (which lack PsbS), and *stn7* plants (which lack Stn7) grown under fluctuating light conditions. The maximum relative growth rate was very similar for the three plant types. Although the fresh weight and final leaf area of *npq4* plants were lower than those of WT and *stn7* plants, the difference was not statistically significant (*p*=0.17 for fresh weight and *p*=0.487 for final leaf area in a comparison of *npq4* with WT). Therefore, no significant disadvantage arising from the absence of Stn7 or PsbS was found under the fluctuating light irradiance condition. Having PsbS or Stn7 also did not confer an advantage under this specific fluctuating light condition (fluctuations are shown in **Figure 1**). The question remained as to whether there is any fluctuating irradiance regime under which a lack of these proteins results in a growth impairment. This has already been shown for *stn7* plants, which show strongly impaired growth under 5 min of low light (50-60 µmol m^-2^ s^-1^) alternating with 1 min of high light (500-600 µmol m^-2^ s^-1^) (Tikkanen et al., 2010; Grieco et al., 2012). The smaller size of the intensity fluctuations (60-360 µmol m^-2^ s^-1^) applied in our light condition was most likely the reason why we did not observe a difference in growth.

We decided to focus in more detail on the need for PsbS, as this protein is directly related to qE and is generally believed to be required for optimal plant growth and fitness under fluctuating light conditions (Kulheim et al., 2002; Alter et al., 2012; Poorter et al., 2016; Vialet-Chabrand et al., 2017; Kaiser et al., 2018; Qiao et al., 2021). In the literature, *npq4* plants have been compared to WT plants under outdoor conditions, and in a climate-controlled room under one specific fluctuating light condition. Here we tested several irradiance fluctuations. Given that temperature might also play a role in the need for qE through its effect on photosynthesis irradiance curves and rate of response to fluctuating light this factor was also included.

#### 3.2.1 What is the effect of fluctuating light on *npq4* plants?

**Figure 6:**
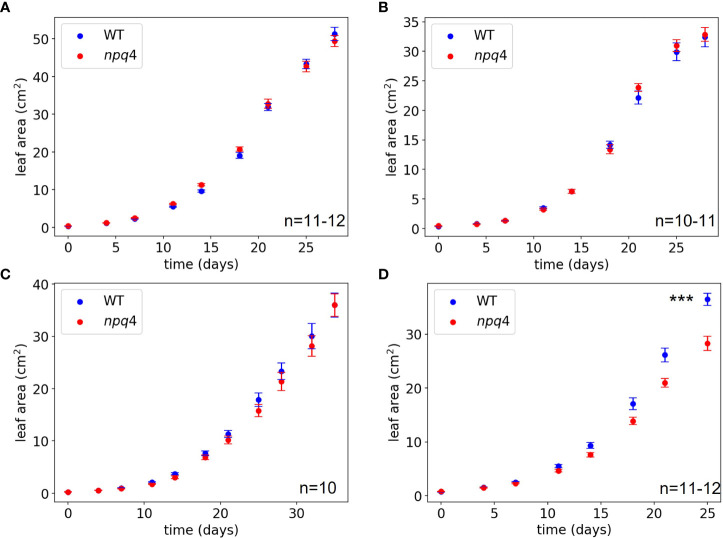
Growth of WT vs npq4 Arabidopsis plants at 24°C under **(A)** continuous light conditions of 125 µmol m^-2^ s^-1^, **(B)** fluctuating light under a regime of 1 hour at 1000 µmol m-2 s-1/0.5 hour 100 µmol m-2 s-1, **(C)** fluctuating light under a regime of 1 min at 1000 μmol m-2 s-1/ 5 min at 100 μmol m-2 s 1. **(D)** fluctuating light under a regime of 5 min at 1000 μmol m-2 s-1/ 5 min at 100 μmol m-2 s 1. The number of plants (n) and SEs are indicated in the figure. Asterisks indicate significant differences in final leaf area, based on a two-tailed independent t-test: *** for p<0.001.

The growth of WT and *npq4* Arabidopsis plants was assessed by quantifying the increase in projected leaf area during growth and their final fresh weight. First, a constant irradiance condition (125 µmol m^-2^ s^-1^) was tested (at 24 °C). As expected, no difference between the WT and *npq4* plants in terms of leaf area (**Figure 6A**) or fresh weight (**Supplementary Figure 2**) was found (**Table 1**). Next, we tested fluctuations occurring on a rather slow timescale: 1 hour of high light (HL, 1000 µmol m^-2^ s^-1^)/0.5 hours of low light (LL, 100 µmol m^-2^ s^-1^), fluctuating during the full 8-hour photoperiod (**Figure 6B**). Again, no difference was found between WT and *npq4*, even though the plants were being exposed to a 10-fold irradiance fluctuation. We continued by testing a higher-frequency fluctuation similar to those used by Tikkanen et al. (2010) and Grieco et al. (2012); specifically, this consisted of 5 min of LL alternating with 1 min of HL (**Figure 6C**; **Supplementary Figure 2**). Even under these light conditions no differences were observed, in agreement with an earlier observation (Tikkanen et al., 2010). This clearly demonstrates that not all intensity fluctuations negatively impact plant growth in the *npq4* mutant. Only when we applied an equal duration of HL and LL by prolonging the exposure to high irradiance (5 min HL /5 min LL) did we find that *npq4* plants showed a significant decrease in growth rate (**Figure 6D**) and fresh weight (**Supplementary Figure 2**) relative to the WT.

**Table 1:** Effects of light conditions on the growth of *npq4* vs WT plants as assessed by leaf area and above-ground fresh weight.

Temperature (°C)	Light conditions during photoperiod (µmol m^-2^ s^-1^)	Decreased growth in *npq4* vs WI
24	125	No
24	1 hour 1000/0.5 hour 100	No
24	1 min 1000/5 min 100	No
24	5 min 1000/5 min 100	Yes
10	125	No
10	5 min 1000/5 min 100	Yes
10	5 min 600/5 min 100	Yes
4	100	No
4	5 min 1000/5 min 100	Yes

**Figure 7:**
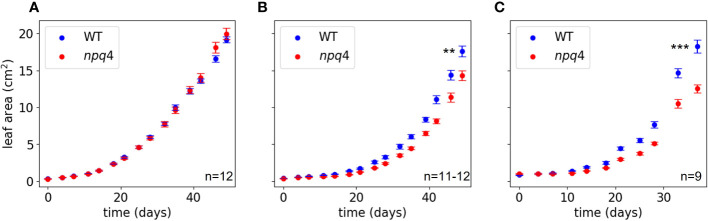
Leaf area in WT and npq4 plants grown at 10 °C under continuous light **(A)**, a regime of 5 min HL (600 µmol m^-2^ s^-1^)/5 min LL **(B)**, and a regime of 5 min HL (1000 µmol m-2 s-1)/5 min LL **(C)**. Asterisks indicate a significant difference in final leaf area, based on a two-tailed independent t-test: ** for p<0.01 and *** for p<0.001.

**Figure 8:**
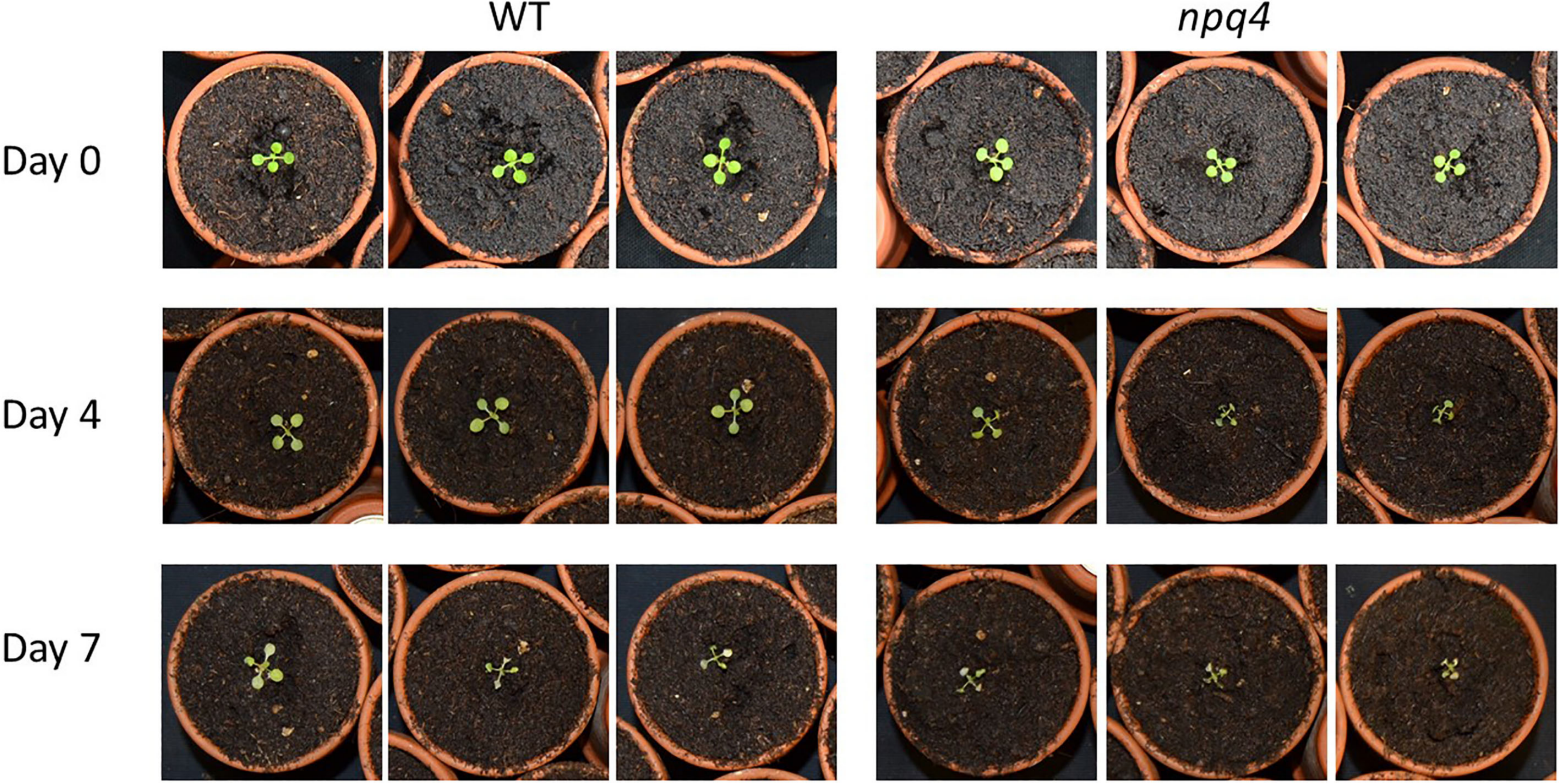
Growth of WT and *npq4* plants under fluctuating light conditions (5 min 1000 µmol m-2 s-1/5 min 100 µmol m^-2^ s^-1^) at 4°C. Number of days after the start of the light treatment is indicated.

We then further challenged the plants by lowering the temperature to increase the light stress. At 10°C, WT and *npq4* plants showed the same amount of growth under continuous light (**Figure 7A**; **Table 1**), although this growth was diminished compared to the same conditions at 24°C. At 10°C, under a regime of 5 min HL (600 µmol m^-2^ s^-1^)/5 min LL (**Figure 7B**) and under a regime of 5 min HL (1000 µmol m^-2^ s^-1^)/5 min LL (**Figure 7C**), *npq4* growth was diminished relative to WT, and this diminution was stronger at the higher HL intensity applied. Comparison of the leaf area of WT plants relative to *npq4* plants (leaf area ratio: WT/*npq4*, **Supplementary Figure 3**) showed that the disadvantage associated with a lack of PsbS is more severe at 10°C compared to 24°C. Decreasing the temperature further to 4°C resulted in half of the *npq4* plants dying after 4 days of exposure to fluctuating light (5 min HL/5 min LL), while 91% of the WT plants survived (**Figure 8**, n≥10). After 7 days, nearly all plants had died in the case of both WT and *npq4*. This indicates that PsbS increases the chance of survival under low temperature conditions, although WT plant mortality was still high.

#### 3.2.2 Improved CO_2_ fixation under fluctuating light

Among the three fluctuating light conditions under which we compared plant growth of WT and *npq4* at 24°C, only the 5* min* HL/5 min LL regime had a negative impact on *npq4* relative to WT. This shows that *npq4* plants, despite their lack of qE, can grow as well as the WT under continuous light or moderate fluctuations (**Figures 6A–C**, **7A**), but there is a growth penalty under harsher treatments involving more rapid fluctuations with longer periods of high irradiance (**Figure 6D**) or fluctuations at lower temperatures (**Figures 7B, C**). The question remains as to whether there are conditions under which the possession of PsbS is a disadvantage—in other words, whether the amount of PsbS is the result of optimization of a trade-off. For instance, it has been shown that tobacco plants lacking PsbS have more open stomata, which decreases the stomatal limitation on CO_2_ assimilation, allowing (all other things being equal) for more assimilation. If water were not a limiting factor and the water vapor pressure deficit only small, then the penalty in terms of plant water balance of having more open stomata would be small. In the case of tobacco, however, despite the increased stomatal conductance arising from knock-out of PsbS, complementary changes in photosynthetic capacity and in the amount of rubisco and its activation left the overall assimilation rate almost unchanged (Glowacka et al., 2018). Furthermore, when WT plants transition from HL to LL, the dissipation of excess energy in the PSII pigment bed through qE does not switch off instantaneously, and as result qE activity limits photosynthesis, wasting potentially useful energy in the PSII pigment bed as heat (Zhu et al., 2004; De Souza et al., 2022). In absence of PsbS, this problem of diminished photosynthesis arising from the slow relaxation of qE ought not to apply, which could make *npq4* more photosynthetically efficient in the immediate aftermath of a high-to-low light transition. Indeed, faster relaxation of qE (which could be achieved by undertaking lower levels of qE to start with) results in greater growth in tobacco plants (Kromdijk et al., 2016), but not in Arabidopsis (Garcia-Molina and Leister, 2020). An obvious disadvantage of lacking PsbS is the increased risk of photodamage under at least some HL conditions. To address these effects on carbon dioxide fixation and photodamage, we explored how the operating efficiency of PSII electron transport (Φ_PSII_) and carbon dioxide fixation were affected by different fluctuating light treatments.

**Figure 9:**
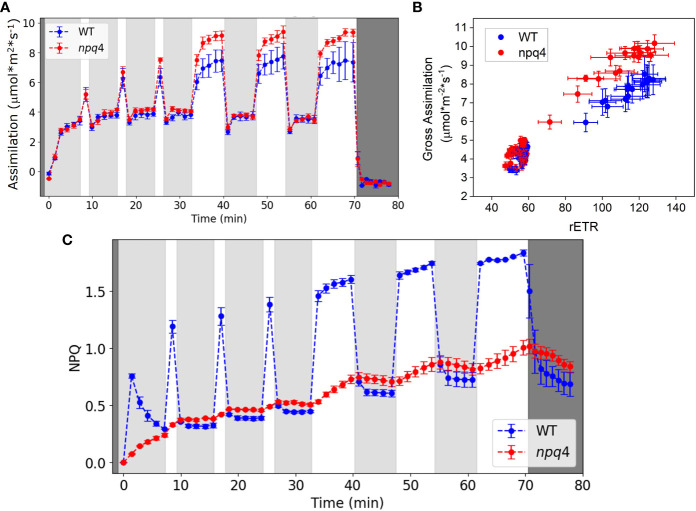
**(A)** Net CO_2_ assimilation in WT and *npq4* plants under fluctuating light conditions. Light gray areas indicate periods of low light (100 µmol m^-2^ s^-1^), white areas indicate high light (1000 µmol m-2 s-1), and dark gray indicates darkness. **(B)** Relationship between gross CO_2_ assimilation and the relative electron transport rate (rETR) of photosystem II. **(C)** NPQ in WT and npq4 plants under fluctuating light conditions. SEs are indicated; n=3. Plants were grown in continuous light.

First, we compared the CO_2_ assimilation rate (**Figure 9A**) and Φ_PSII_ (**Supplementary Figure 4**) of WT and *npq4* plants under fluctuating light conditions. Dark-adapted plants, grown under constant light, were exposed to three cycles of approximately 5 min LL/1 min HL, followed by three cycles of 5 min LL/5 min HL. A portable gas exchange system (LI-6400XT) equipped with red and blue actinic light was used for these measurements. Under these conditions, CO_2_ assimilation during the 5 min of HL was higher for *npq4* plants than for WT plants. To explore the cause of this enhanced assimilation, we plotted gross CO_2_ assimilation rate (i.e., the assimilation rate referenced to the respiration rate during the dark period after the end of the photorespiratory burst and other short-lived transients occurring after the cessation of irradiance) against relative electron transport rate (rETR) through photosystem II. rETR, an index for the rate of linear electron transport, is obtained by multiplying Φ_PSII_ by the light intensity, assuming that leaf absorption is the same for WT and *npq4* plants (**Figure 9B**). *npq4* plants showed a higher assimilation rate per unit rETR (*p*<0.0001, two-tailed test). This might be explained by a higher internal CO_2_ concentration in *npq4* plants, consistent with their larger stomatal conductance as compared to WT plants (**Supplementary Figure 5**).

In **Figure 9C**, the NPQ levels of WT and *npq4* plants are compared under the same fluctuating light cycles used to investigate the assimilation responses. As expected, WT plants showed qE under HL, and this was lower in *npq4* plants. Despite lacking PsbS, however, the *npq4* plants did develop a substantial level of NPQ during the experiment. qE in the *npq4* plants tracked qE in the WT plants during the low light periods but increased only slowly during the high light periods. The slow increase of NPQ in *npq4* plants during the high light periods paralleled the slow increase in NPQ in WT plants under high light. NPQ levels during the LL illumination periods were even slightly higher in *npq4* plants than in WT plants. Although they do have a limited, slow NPQ response, the *npq4* plants lack the large and rapid NPQ response of the WT plants that can be seen immediately following the beginning and end of the HL periods.

**Figure 10:**
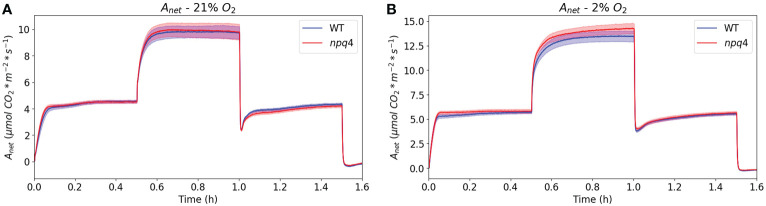
Net CO_2_ assimilation in WT and *npq4* plants under a regime of 30 min LL – 30 min HL – 30 min LL with 21% oxygen **(A)** and 2% oxygen **(B)**. SEs are indicated; n=9. Plants were grown in continuous light.

Next, to assess CO_2_ assimilation directly after a switch from HL to LL in greater detail, we exposed WT and *npq4* plants to a regime consisting of 30 min of LL/30 min HL/30 min LL under atmospheric (21%) oxygen levels (**Figure 10A**). In this case, data were collected using a custom-built gas analysis system with white actinic LEDs; this enabled the measurement of the assimilation of a single Arabidopsis leaf. Under these conditions, similar assimilation rates were measured for WT and *npq4* leaves. Upon transition from HL to LL, the drop in assimilation was also very similar for both types of plants, so we did not observe any advantage associated with a lack of PsbS in the period immediately following a switch from HL to LL. The drop in net assimilation after a switch to LL is partially attributable to the CO_2_ burst that occurs following the high-to-low irradiance step, which itself is due to photorespiratory carbon dioxide release (Vines et al., 1983). To remove this feature from the assimilation response, we reduced the oxygen level to 2% (**Figure 10B**). However, even without photorespiration and qE (*npq4*), a post-high-irradiance drop in assimilation was still observed to some extent after the transition to LL, although the time course of the transient post-illumination drop in assimilation was slower under non-photorespiratory (2% O_2_) conditions compared to photorespiratory conditions. The effect of removing qE (as observed through comparison of *npq4* with WT) on this post-high-light drop is limited (**Figure 10B**).

An obvious disadvantage that *npq4* plants can be expected to have relative to WT plants is their increased risk of photodamage in HL conditions. We used the maximum quantum efficiency of PSII (F_v_/F_m_) as a proxy for the degree of photodamage. Plants were grown for 5 days under a fluctuating regime of 5 min HL/5 min LL. After growth at 24°C, F_v_/F_m_ was significantly (although only slightly) lower in *npq4* plants (0.803 ± 0.003; ± indicates the standard error) compared to WT plants (0.822 ± 0.002); see **Supplementary Figures 6A, B**. However, when the temperature was 10°C, the difference was larger: 0.770 ± 0.006 for WT vs 0.710 ± 0.006 for *npq4* (**Supplementary Figure 6C**). This indicates that PsbS protects PSII against photodamage under this more extreme fluctuating light treatment.

### 3.3 Improved CO_2_ fixation under parabolic light

**Figure 11:**
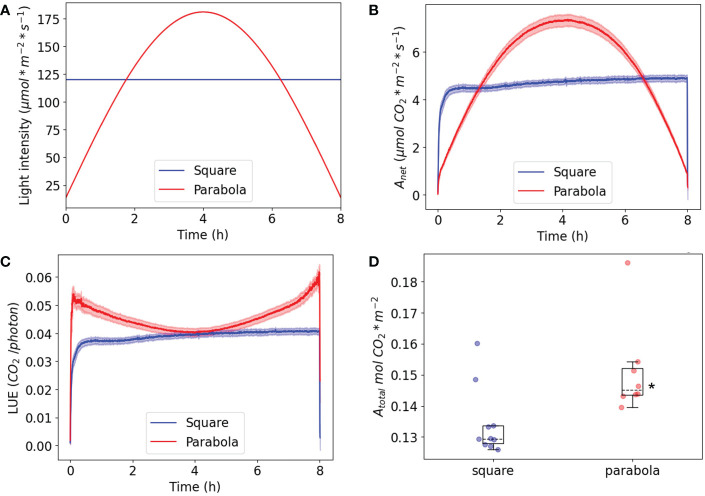
Plants were grown and measured under a square-wave irradiance profile and a parabolic irradiance profile with the same daily integral PPFD. **(A)** The irradiance profile during the 8h measurement. **(B)** The net CO_2_ assimilation rate. **(C)** The net light-use efficiency (LUE). **(D)** The total CO_2_ assimilation per day. The SE is shown; n=8 for the parabola condition, n=10 for the square condition. The asterisk is D indicates a significant difference based on a two-tailed independent t-test (p< 0.05).

The question remains as to why plants grow faster in parabolic light. Given that plants have evolved under conditions in which light intensity naturally increases and decreases gradually during the day, it can be hypothesized that short- or longer-term control of net carbon assimilation rate and stomal conductance has been in some way optimized for the conditions. To investigate this possibility, plants were grown under square-wave irradiance (PAR: 125 μmol m^-2^ s^-1^), after which they were tested under square-wave white light (120 µmol m^-2^ s^-1^) or parabolic white light (minimum PAR of 15 µmol m^-2^ s^-1^; maximum of 180 µmol m^-2^ s^-1^) with the same integral PPFD (**Figure 11A**). Carbon assimilation rates (**Figure 11B**) and transpiration rates (**Supplementary Figure 7**) were measured over an entire photoperiod. For square-wave light, carbon assimilation rate rapidly rose and reached its maximum after ~20 minutes, after which it slowly and slightly increased over the remainder of the photoperiod. The parabolic irradiance profile showed a gradual increase and decrease, following the light intensity pattern (**Figures 11A, B**). The most interesting data were obtained by dividing carbon assimilation rate by light intensity as an index of light-use efficiency (LUE) (**Figure 11C**). For the plants grown and tested under parabolic light conditions, LUE reached its maximum value in under 2 minutes, while this took 20 minutes for the plants tested under square-wave light conditions (see **Supplementary Figure 8** for a zoomed-in visualization of the first hour). Furthermore, the LUE of plants exposed to a parabolic irradiance profile was higher at the beginning and end of the day, when the leaves received a lower intensity of light compared to the plants exposed to the square-wave condition. This is to be expected, as gross CO_2_ assimilation LUE is known to be highest under low-light conditions (Bjorkman and Holmgren, 1963). Interestingly, the LUE of plants grown under a parabolic irradiance profile was the same in the middle of the day as that of plants grown under a square-wave irradiance profile, while the light intensity at midday was 180 µmol m^-2^ s^-1^ for the parabolic profile and 120 µmol m^-2^ s^-1^ for the square-wave profile. Taken together, these findings indicate that, over the course of a full day, light presented with a parabolic profile can be used more efficiently; this finding also implies that Arabidopsis plants acclimate their photosynthesis processes differently to each of the two regimes, resulting in the same light-use efficiency at the peak irradiance of both regimes.

To evaluate further this apparent improvement in assimilation in response to a parabolic irradiance profile, total CO_2_ assimilation (mol CO_2_ m^-2^) per day was compared for the two growth conditions (**Figure 11D**). This comparison showed that the parabolic irradiance profile resulted in significantly higher levels of total assimilation per day than the square-wave profile (n≥8), even though the daily integral of irradiance was the same. This is partly due to the parabolic profile containing lower irradiances than the square-wave profile, since lower irradiances will be associated with higher light-use efficiency in terms of assimilation, all other things being equal. It is also due to the parabolic profile producing higher light-use efficiency than the square-wave profile at higher irradiances, thereby enabling the plants to make better use of the higher irradiances. The higher daily integral of assimilation can explain why the WT plants grown under the parabolic irradiance profile had a significantly higher fresh weight and exponential growth rate (**Figure 4**) compared to the plants grown under a square-wave profile.

## 4 Discussion

### 4.1 Plants under natural light conditions

Over recent decades, photosynthesis research has focused on photosynthesis under constant light conditions. However, in nature and for crops that grow in the field, light is essentially never constant, and under these circumstances photosynthetic responses to fluctuating light become more important (Harbinson and Woodward, 1984; Pearcy, 1990; Ruban, 2009; Kaiser et al., 2018; Wang et al., 2020; Long et al., 2022). When plants transition from shade into full sunlight, absorbed irradiance can increase over the sub-second time range, but the reactions of photosynthesis, especially the dark reactions and stomatal responses, take many minutes to reach new steady-state levels, with the slowest phases of this response being limited by the rates of rubisco activation and stomatal opening (Allen and Pearcy, 2000; Mott and Woodrow, 2000; McAusland et al., 2016; Kaiser et al., 2018; De Souza et al., 2020). This relatively slow increase in the rate of photosynthesis leads to the loss of potential canopy CO_2_ assimilation (Taylor and Long, 2017). Furthermore, the excess of light energy that is harvested by the plant may lead to photodamage, particularly in PSII, due to the formation of reactive oxygen species. To minimize photodamage, plants rapidly upregulate qE in order to safely thermally dissipate the excess energy (Horton et al., 1996). When plants shift from sunlight into the shade, however, qE takes on the order of minutes to relax to a new lower level. As a result, useful excitation energy is wasted, and assimilation is transiently limited by the depressed light-use efficiency of PSII photochemistry, which lowers the potential carbon gain (Zhu et al., 2004; De Souza et al., 2022). There is, therefore, an intriguing trade-off between photoprotection when transitioning to a high irradiance and wasting energy when transitioning to a low irradiance. It can be hypothesized that in conditions of overall low light with brief spikes of high light (on a seconds-to-minutes timescale, typical for sun flecks), it is more efficient to endure photodamage in high light rather than switching to a dissipative state that does not instantly switch off in the period after the spike.

When the periods of high light are as long as the periods of low light, on a timescale of minutes, the prolonged exposure to excess light means that inducing qE could be more worthwhile in order to reduce photodamage and thus allow for increased CO_2_ assimilation as a result of higher LUE and eventually greater plant growth. This benefit of qE over longer periods of high light would be in spite of the loss of carbon assimilation that occurs immediately after the high–low light transition due to the slow relaxation of qE. The following questions arise: first, under which light conditions does photoprotection represent an advantage for plant growth, and under which conditions it is not beneficial and may it even impair plant growth?; and second, is a light intensity profile involving a gradual increase and decrease during the day better for plant growth than a square-wave irradiance profile, of the kind often used in growth cabinets? To answer these questions, we have grown plants under various light conditions and compared their increase in leaf area and biomass production. These growth conditions made use of rather unnatural square-wave profiles of the kind that are nonetheless widely used in fluctuating light research, as well a fluctuating light profile recorded in a maize canopy and an approximately parabolic profile that is similar to the natural diurnal daylight profile of a cloudless sky. WT plants were compared with *npq4* plants, which lack the PsbS protein that is key to the qE component of NPQ, and *stn7* plants, which are impaired in their regulation and optimization of light-harvesting *via* state transitions.

### 4.2 When does PsbS represent an advantage?

Comparing the growth of WT vs *npq4* Arabidopsis plants under various fluctuating light conditions at controlled temperatures (24°C, 10°C, and 4°C) allowed us to investigate the circumstances under which having PsbS represented an advantage. The plants were well watered and fertilized to ensure that water and nutrient stress would not compound the effects of light and temperature stress. First, the effect of a natural fluctuating light profile (**Figure 1**), measured under a maize canopy, was tested. No significant difference was found between WT and *npq4* plants in leaf area or fresh weight (**Figure 5**). Alternating 1h of HL (1000 µmol m^-2^ s^-1^) with 0.5h of LL (100 µmol m^-2^ s^-1^) also resulted in no differences between WT and *npq4* plants. We cannot rule out an effect of acclimation processes, such as a decrease in PSII antenna size and the increase in linear electron transport efficiency that occurs when plants are acclimated to HL for several days (Bailey et al., 2004; Albanese et al., 2016; Schumann et al., 2017; van Rooijen et al., 2017). Such acclimation could make qE less important for photoprotection. Furthermore, the increased opening of stomata in *npq4* plants could represent an advantage for plant growth (Drake et al., 2013; Glowacka et al., 2018) (**Figure 9B**), while the corresponding natural disadvantage of lower water use efficiency would be less of a problem under our growth conditions, as the plants were well watered (Glowacka et al., 2018). Our measurements did indeed show an increased CO_2_ assimilation rate in *npq4* plants during conditions involving 5 min of high red and blue light illumination, but this advantage was not confirmed under 30 min high white light illumination.

Next, we tested the plant growth response to a regimen of 5 min of LL (100 µmol m^-2^ s^-1^) alternating with 1 min of HL (1000 µmol m^-2^ s^-1^). Again, no difference in plant growth was observed. It can be hypothesized that a certain amount of extra photodamage might be induced in *npq4* plants during the brief period of HL illumination; however, this could be compensated for by the reduced impact of qE on electron transport following a shift to LL. To test whether *npq4* plants do indeed perform better immediately after a shift from HL to LL, we compared CO_2_ assimilation in WT and *npq4* plants. No advantage of a lack of PsbS was observed (**Figure 10**), so this cannot explain why *npq4* plants did not show impaired growth under the regime of 5 min of LL (100 µmol m^-2^ s^-1^) alternating with 1 min of HL (1000 µmol m^-2^ s^-1^). Instead, our NPQ measurements suggest that this can be explained by the higher overall NPQ levels occurring in the *npq4* plants during the periods of LL illumination. It is interesting to see that the level of NPQ during the LL periods is even slightly higher for *npq4* plants than for WT plants and increases on the same trend during each illumination cycle. Additionally, the decay of NPQ in the dark follows a similar trend for *npq4* and WT plants (**Figure 9C**); furthermore, this residual NPQ in *npq4* plants has been shown to largely decay within 15 min (Takahashi et al., 2009). This is clearly a faster process than the PSII repair cycle, which takes hours to complete (Koivuniemi et al., 1995) and includes a D1 degradation half-time of ~30 min (Aro et al., 1993). As such, the NPQ that has developed in *npq4* plants appears to be largely photoprotective (that is, like qE) and occurs only in part due to permanent PSII damage. This conclusion is in agreement with the work of Johnson and Ruban (2010), who showed that photoprotective energy dissipation does build up in the *npq4* mutant, albeit with a far slower kinetics.

Finally, alternating 5 min of HL with 5 min of LL did result in better growth in WT plants, signifying that having PsbS represents an advantage under this fluctuating light regime. Analysis of dark-acclimated F_v_/F_m_ after one week of this fluctuating light regime showed that dark-adapted quantum efficiency of PSII was lower for *npq4* plants than for WT plants. This effect was stronger at 10°C than at 24°C. This shows that PsbS plays a photoprotective role that becomes more important at lower temperatures under more extreme fluctuating light conditions (**Figures 6D**, **7B, C**). This could be due to a lower rate of PSII repair, or to a larger PSII acceptor-side limitation upon shift from LL to HL, as the induction of photosynthesis will be slower (Tikkanen et al., 2014; Huang et al., 2021). In agreement with this observation, we found that the combination of this fluctuating light regime with a lower temperature of 10°C increased the difference in plant growth between WT and *npq4* plants. Furthermore, at 4°C, *npq4* seedlings died after 4 days of exposure to the fluctuating light regime, while nearly all WT seedlings survived. This points to a clear survival benefit if Arabidopsis plants were to be exposed to cold days with high light levels, as might occur in late autumn, winter, and spring (Arabidopsis is a winter annual over much of its European distribution).

In nature, stresses often occur together. For example, on a summer day, high irradiance can be accompanied by high temperatures and drought stress. Examining individual factors in isolation is essential in order to understand their effects in detail; however, this approach might not capture the actual conditions in nature. With the increasing opportunities for high-throughput phenotyping, the time is ripe to study the combined effect of heat, drought, and light stress under controlled conditions. The disadvantage of this approach is that the number of possibilities to be tested is infinite. It is therefore important that the research community selects specific conditions to be tested. To investigate the role of PsbS in photoprotection, we recommend including a regime consisting of the alternation of 5 min of high light with 5 min of low light, which shows a clear phenotype for *npq4* plants. Given that the major component of PsbS-dependent NPQ is activated over the course of minutes and deactivated on a timescale of tens of seconds (Li et al., 2000), it would also be interesting to investigate whether these more rapid fluctuations have a greater impact on *npq4* vs WT plant growth. Past research on the effects of fluctuating light on growth have been largely limited by the ability to modulate growth room irradiance. In most cases, irradiance could only be modulated at low frequencies and amplitudes (compared to those of natural irradiances, which can reach 2,000 μmol m^-2^ s^-1^). With the advent of LED lighting in controlled environments and the higher irradiances that can be produced by LEDs, it will become possible to explore a wider range of frequencies and amplitudes of irradiance. Given the complexity of the regulation of photosynthesis, with which qE regulation is interwoven, and the way in which photodamage arises from this regulation, a question that emerges is which frequencies and amplitudes of irradiance result in the greatest negative impact on plant growth, especially in a comparison between *npq4* and WT. For example, in regard to photodamage and fluctuating light, other targets exist apart from PSII; e.g., PSI has also been shown to be vulnerable to photodamage at low temperatures, and this damage is influenced by qE in PSII (Zhang and Scheller, 2004; Tikkanen et al., 2014; Allahverdiyeva et al., 2015). A valuable aspect of examining effects on plant growth as a way to monitor factors like the absence of PsbS is that this approach integrates across different sources of stress and will include any positive consequences of an apparently deleterious mutation on growth.

### 4.3 Gradual day/night regime improves plant growth by more efficient photosynthesis

Recent advances in LED technology have made this the primary choice of light source for vertical farming and for growth cabinets used for scientific research. The favorable characteristics of LEDs include low energy requirements (or high quantum efficiency), a low heat output, fast response time, and flexibility in light intensity, with attendant wide variation in the spectral composition of the irradiance that can be produced (Proietti et al., 2021; van Delden et al., 2021). When plants are grown under artificial light, it is important that the energy input is efficiently converted into crop yield. In this project, we used the controllability of LED output to simulate a natural diurnal pattern of increasing and decreasing light intensity in order to investigate whether this would lead to higher LUE, which would be of interest for vertical farming. In the case of WT Arabidopsis plants grown under a parabolic irradiance profile, we did indeed observe an increase in maximal exponential growth rate and final fresh weight in comparison to those grown under a square-wave profile (**Figure 4**). Further investigation of LUE in terms of assimilation (**Figure 9**) showed that the low-irradiance part of the parabolic profile occurring at the beginning of the day can be used very efficiently and efficiency rises quickly with duration of irradiance. This indicates that assimilation is hardly limited by photosynthetic induction (particularly rubisco activation and stomatal opening). On the other hand, the sudden increase of irradiance in the square-wave regime results in a low LUE for the first ~20 minutes because of the time required to strongly activate assimilation. It remains to be seen whether a slow onset of light intensity in combination with stepwise switching off of the light (at the end of the photoperiod) provides the same increase in biomass and total CO_2_ assimilation, or whether the natural gradual rise and decrease in light intensity provides the highest overall LUE.

## Data availability statement

The original contributions presented in the study are included in the article/**Supplementary Material**. Further inquiries can be directed to the corresponding authors.

## Author contributions

CS, JH, and EW contributed to conception and design of the study. LC designed and built the system to grow plants under fluctuating light. CS, LC, KS, CS, TT, and SD performed the experiments. CS organized the data, made the figures and performed the statistical analysis. EW and CS wrote the draft of the manuscript, SD and JH substantially improved the manuscript. All authors contributed to the article and approved the submitted version.
