# Supplementary material for: The effects of different daily irradiance profiles on Arabidopsis growth, with special attention to the role of PsbS
Source: Front Plant Sci. 2023 Mar 9;14:1070218. doi: 10.3389/fpls.2023.1070218 (PMC10035889; doi:10.3389/fpls.2023.1070218)
Supplement: Supplementary file 1 [file DataSheet_1.docx]

Supplementary Material

## Supplementary Movies


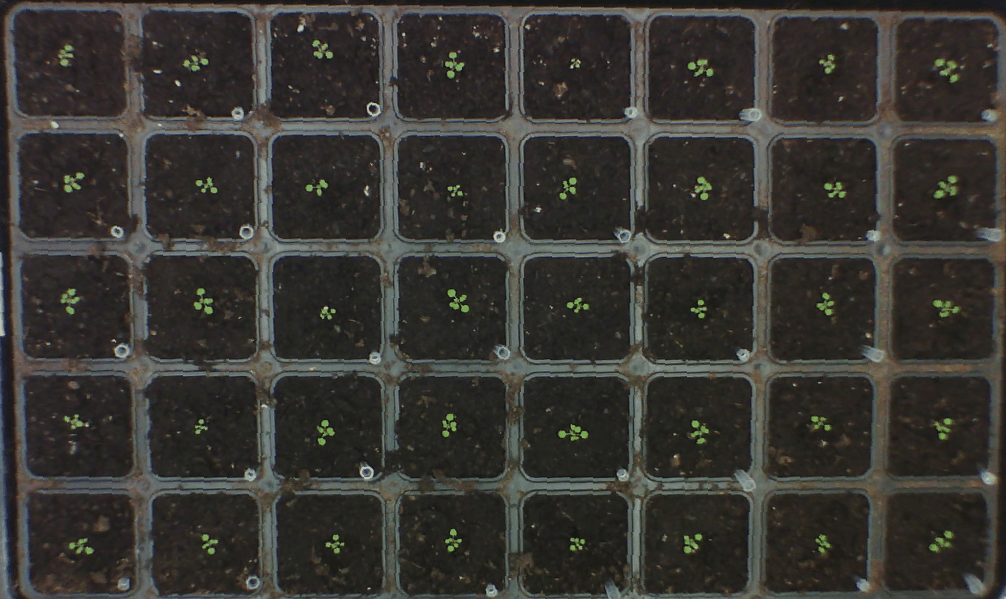


***SI Movie 1.*** *Photographs of plant growth.*

***
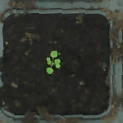

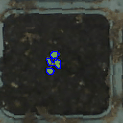

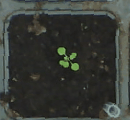

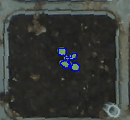
***

***SI Movie 2.*** *Automatic selection of leaf area.*

## Supplementary Figures


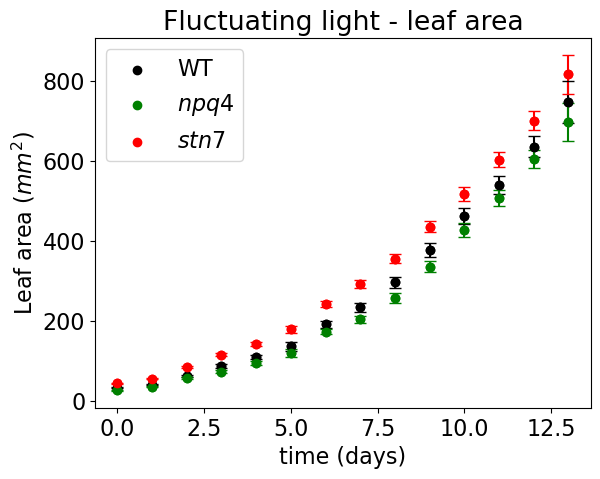


***SI Figure 1.*** *Leaf area of WT, npq4 and stn7 plants grown under fluctuating light (see Fig. 1 of manuscript for the fluctuations).*


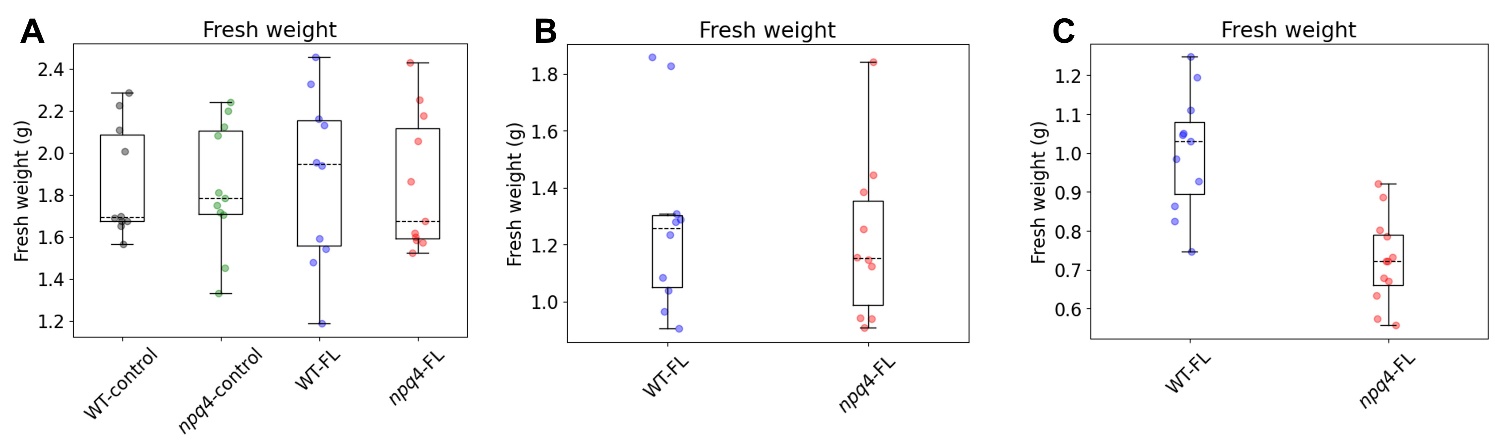


***SI Figure 2.*** *Comparison of fresh weight of WT and npq4 plants grown under 125 µmol photons m^-2^ s^-1^ continuous light (control) and or 1h HL/ 0.5h LL fluctuating light (FL) (A), 1 min HL/ 5 min LL (B), 5 min HL/ 5 min LL (C).*

***
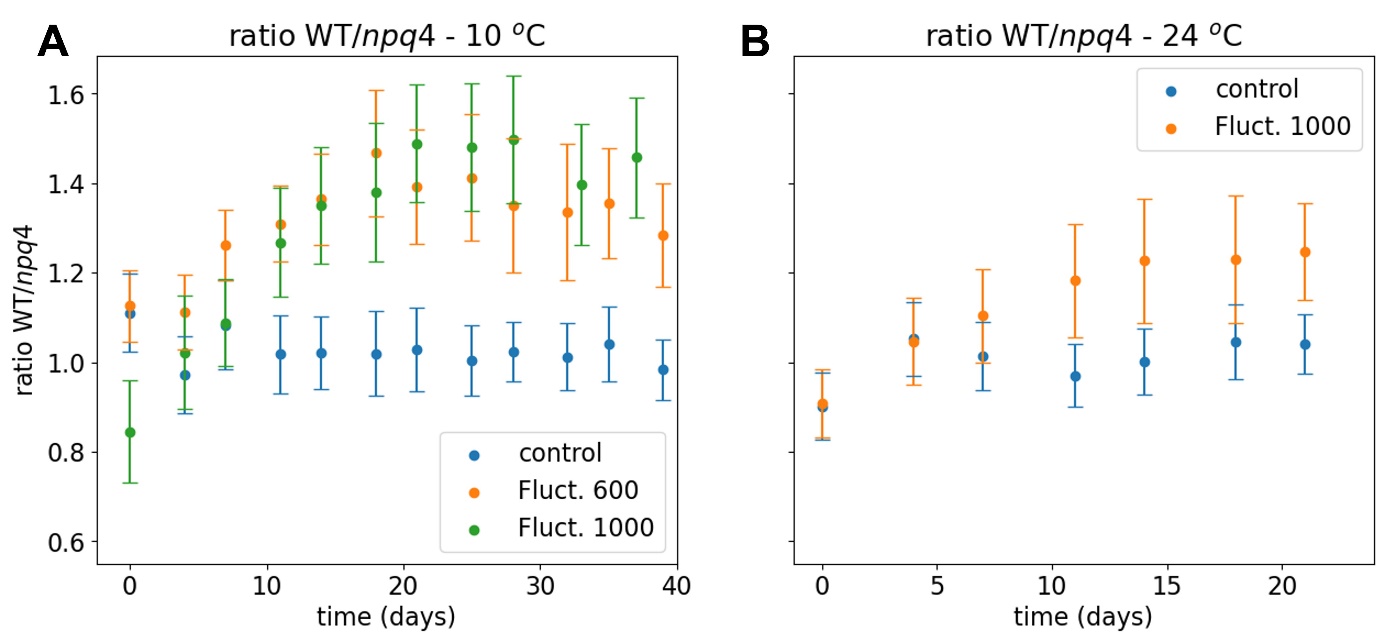
***

***SI Figure 3.*** *Ratio of leaf area for WT vs npq4 plants grown under fluctuating light: 5 min HL (600 µmol photons m^-2^ s^-1^)/ 5 min LL and 5 min HL (1000 µmol photons m^-2^ s^-1^)/ 5 min LL at 10 ^o^C (A), and 5 min HL (1000 µmol photons m^-2^ s^-1^)/ 5 min LL at 24 ^o^C (B).*

*
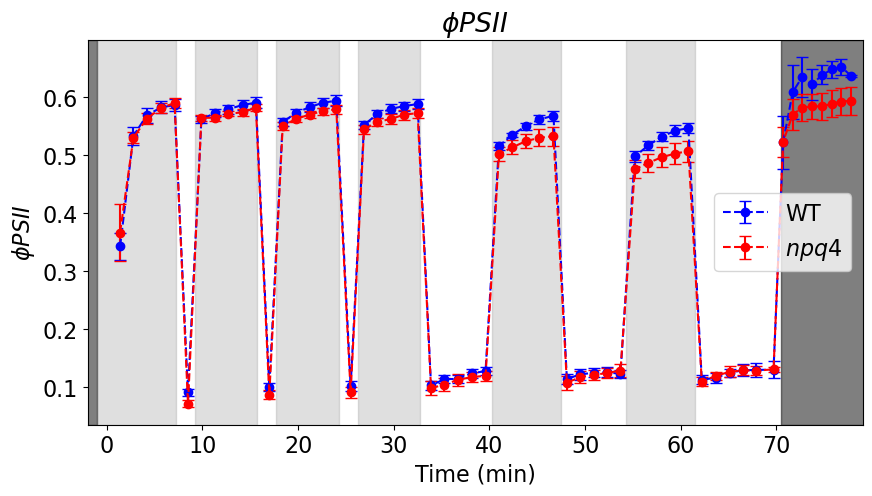
*

***SI Figure 4.*** *PSII operating efficiency for WT and npq4 plants under fluctuating light: three cycles of approximately 5 min. LL (white)/ 1 min. HL (grey), followed by three cycles of 5 min. LL (white)/ 5 min. HL (grey). Note that in the dark grey area (darkness) it is technically speaking not the PSII operating efficiency. SE is shown, n=3.*

*
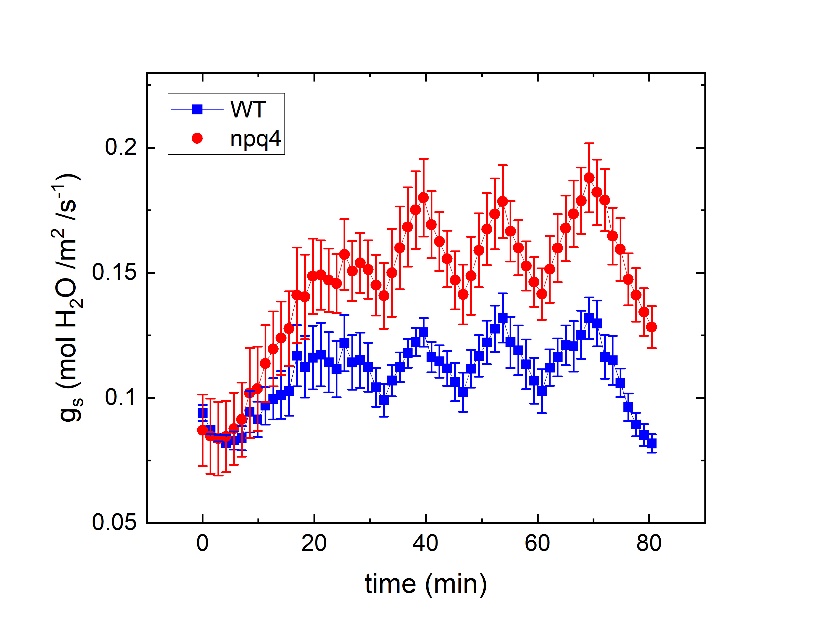
*

***SI Figure 5.*** *Stomatal conductance of WT and npq4 plants under fluctuating light: : three cycles of approximately 5 min. LL/ 1 min. HL, followed by three cycles of 5 min. LL/ 5 min. SE is shown, n=3.*

*
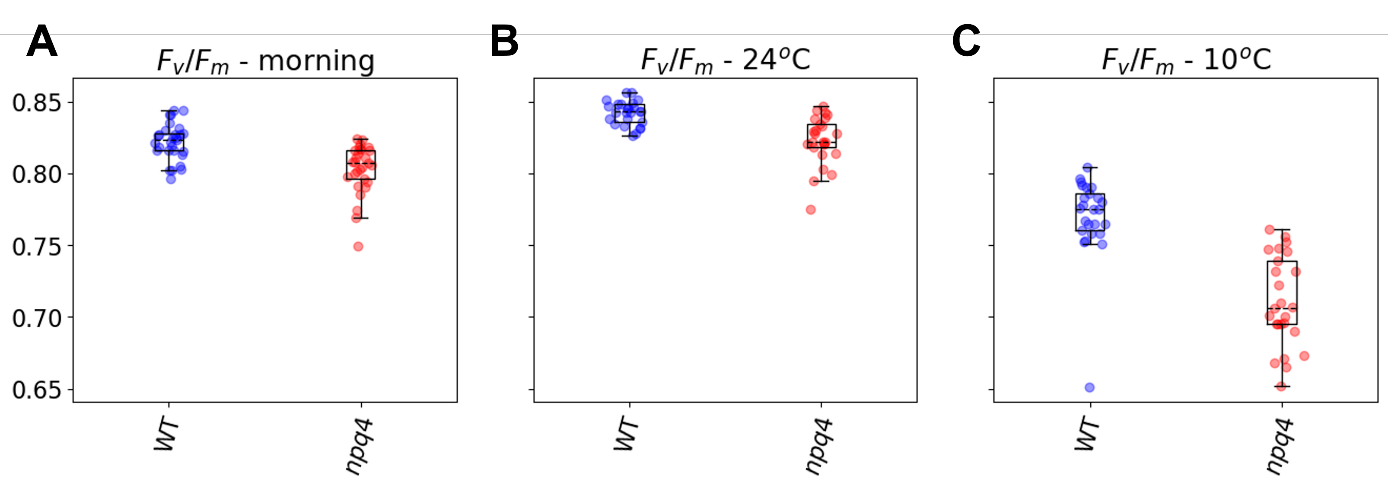
*

***SI Figure 6.*** *Fv/Fm values of WT and npq4 plants exposed for 7 days to 5 min. HL/ 5 min. LL during the photoperiod of 8h. A. measurement in the morning, before the start of the photoperiod, 24 ^o^C growth temperature. B. Measurements after 5h of exposure to fluctuating light followed by 30 min. dark adaptation, 24 ^o^C growth temperature. C. Measurements after 5h of exposure to fluctuating light followed by 30 min. dark adaptation, 10 ^o^C growth temperature. WT and npq4 plants have significantly different values, One-way ANOVA, p<0.05, n ≥ 25.*


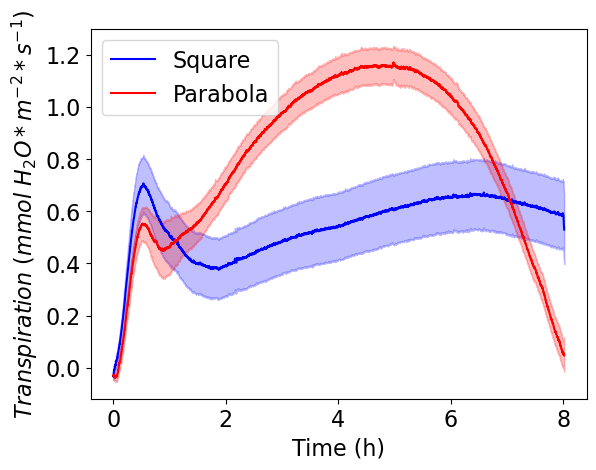


***SI Figure 7.*** *Transpiration rate of WT plants grown and measured under square and parabolic light.*

*
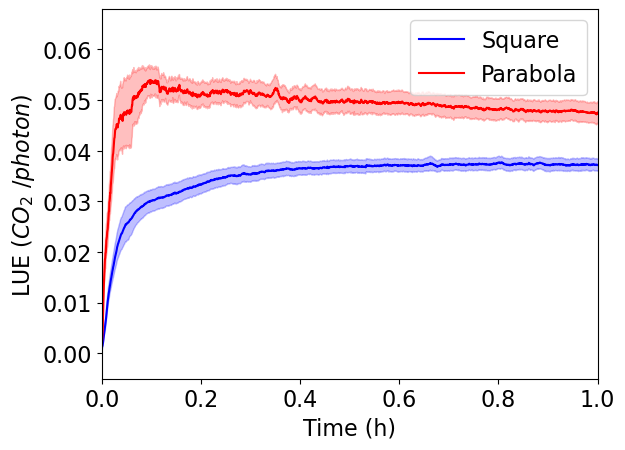
*

***SI Figure 8.*** *Zoom in of light-use efficiency (LUE) curve for plants tested under square wave and parabolic light.*
